# Book Notices

**Published:** 1874-03

**Authors:** 


BOOK NOTICES.—We promised and desired to notice several valuable books in this issue, but wishing to give something more than mere notices, in order to properly bring their merits before our readers, we are compelled to let them lie over until our next.

